# José Baselga M.D., Ph.D. (1959–2021) leading cancer researcher and oncologist

**DOI:** 10.1186/s13046-021-01966-y

**Published:** 2021-05-07

**Authors:** Moshe Elkabets, Giovanni Blandino

**Affiliations:** 1 The Shraga Segal Department of Microbiology, Immunology and Genetics Faculty of Health Sciences , Ben-Gurion University of the Negev , Beer-Sheva, 84105 Israel; 2 Oncogenomic and Epigenetic Unit, IRCCS, Regina Elena National Cancer Institute, Rome, Italy

On March 21st, 2021, one of the most remarkable leaders in oncology Dr. José Baselga – friend, colleague and mentor – passed away before his time. The entire cancer research and oncology community was shocked by the premature death of José, who for three decades led the therapeutic revolution towards personalized/precision medicine using targeted therapies. José’s ability to envisage treatment in the years ahead led him to become one of the best-known leaders in drug development in medical oncology.

At the age of 27, after graduation from the University of Autonoma de Barcelona, military service duty, and a year of residency at Vall d’Hebron hospital in Barcelona, José moved to New York to start his career as a breast cancer oncologist and researcher. By day, José dedicated his time to treating cancer patients at Kings County Hospital in Brooklyn and by night and on holidays, to exploring new treatments in the lab at Memorial Sloan Kettering (MSK). José was especially intrigued by the molecular mechanisms of the response to anti-cancer therapies, and this curiosity and hunger for knowledge accompanied him throughout his extraordinary career.

In 1989, at the age of 30, José moved to MSK to work in the clinic with Larry Norton and in the laboratory with John Mendelsohn to investigate the anti-tumor activity of therapies that block the epidermal growth factor receptor (EGFR) and HER2 on cancer cell lines. In the lab, José demonstrated the relevance of blocking EGFR in lung and breast cancer [1–3], and in a work that was published in JNCI 1993, he provided the rationale for combining an anti-EGFR antibody therapy (cetuximab) with chemotherapy. In addition, he demonstrated, in the lab, how overexpression of HER2 limited the efficacy of chemotherapies in breast cancer [4, 5]. As a resident, José led many clinical trials and showed the potential of targeting HER2 in breast cancer patients with HER2 overexpression and of targeting EGFR using cetuximab in EGFR-overexpressing tumors [6–8]. José’s achievements in designing and running clinical trials and in performing translational research laid down the grounds for his subsequent career as a prominent oncologist and drug developer.

In 1996, José became the Chairman of Medical and Radiation Oncology and Hematology at Vall d’Hebron Hospital in his hometown, Barcelona, and ten years later, he founded the Vall d’Hebron Institute of Oncology (VHIO). As a director of VHIO, José furthered his vision of promoting translational research by building the bed-to-bench culture. To this end, he hired extremely talented basic researchers, supervised them to become translational researchers, and trained medical oncologists to deliver science-driven trials. Joan Albanell, Miguel Angel Molina and José Tabernero were a few of many talented first generation of mentees who worked with José at Vall d’Hebron. The impressive clinical and translational research activities at VHIO built the reputation of the center and that of José in concert. Between 2000 and 2010, José led or was involved in over 110 publications of Phase I and Phase II studies and 17 publications of Phase III studies. Among others, worth mentioning are four published in the New England of Medicine on therapy combinations with anti-HER2 or cetuximab in breast and head and neck cancers, respectively—combinations that were later approved by the FDA [9–12] and saved the lives of thousands of cancer patients. José was always at the frontline of research on new molecular targets, and in addition to anti-EGFR and HER2 therapies he became interested in those blocking the PI3K/mTOR pathway, which regulates tumor progression. Side by side with the development of pan-PI3K/mTOR inhibitors in the clinic, José nurtured a second generation of translational investigators, among them Maurizio Scaltriti and Violeta Serra. The research team in Barcelona worked to elucidate mechanisms of resistance to anti-HER2 and PI3K therapies and demonstrated novel concepts of re-activation of survival signaling pathways [13–18]. Their findings provided the rationale for testing therapeutic combinations such as concomitant PARP and PI3K inhibition. On the clinical side, José was involved in the training of accomplished oncologists like Jordi Rodon, Cristina Saura, and Javier Cortes.

In early 2000 Josè was a member of the International Advisory Board of Regina Italian National Cancer Institute in Rome and his unique scientific impetous led to the implementation of Disease Team Management. Their multidisciplinary approach is fundamental for the transational and clinical management that rountinely we offer to admitted patients.

After 14 years at Vall d’Hebron, José accepted the position of Chief of Hematology and Oncology at Massachusetts General Hospital (MGH) in Boston. For this next challenge, José encouraged Maurizio Scaltriti to become a Harvard Medical School instructor and build his lab from scratch. At MGH, José and Maurizio recruited the third generation of translational investigators, whose primary focus was to uncover mechanisms of response and resistance to isoform-specific PI3K inhibitors. On the clinical side, Dejan Juric led the clinical development. José, in his charismatic and elegant way, encouraged all group members to join forces and expertise to test anti-PI3K agents in pre-clinical models and to validate the findings in cancer patients [19–22]. The studies generated by José’s lab, together with those of other outstanding leaders in the field, such as Carlos Arteaga, Gordon Mills, Lew Cantley, Jeffery Engelman, Neal Rosen, and Levi Garraway, provided molecular insight on the effectiveness of and resistance to these agents in *PIK3CA*-mutated solid cancers.

In January 2013, José was recruited at Memorial Sloan Kettering Cancer Center (MSKCC) as Physician-in-Chief and relocated his lab to that institution. At MSKCC, José expanded the research on isoform-specific PI3K inhibitors into epigenetics [23–26] and into other pathologies, such as head and neck cancers and venous malformations [27–29]. During the almost 6 years at MSK, José chalked up a few major milestones, including the establishment of the Center for Molecular Oncology (CMO), Early Drug Development Services led by the talented David Hyman. He wanted – and obtained – an in-house targeted genomic sequencing platform (MSK-IMPACT), which would broaden therapeutic alternatives for cancer patients, as it would enable cancer patients to enroll in basket trials based on genomic alterations their cancers. He was thus involved in developing and designing of several basket trials and reinforced the importance of genomic-driven medicine in medical oncology [30–33]. By virtue of these efforts, genomic sequencing and precision medicine became the norm for most cancer patients at MSKCC. The entire scientific community, and José’s research group as part of it, benefited from the massive genomic sequencing information generated at the CMO, and the fourth (and last) generation of translational investigators utilized these data to focus on the underlying role of specific genomic alterations in the response and resistance to multiple treatments in cancer patients [24, 34, 35].

In 2017, Josè was awarded with the Lombroso Prize at the Weizmann Institute of Science in Israel. His award lecture was outstanding. His unique ability to surf into basic, translational and clinical research captured the audience and instigated a number of questions that lasted over the lunch award ceremony.

In September 2018, José resigned from MSK and joined to AstraZeneca (AZ) as the Executive Vice President of Oncology R&D, and started to bring an academic view into industry by accelerating clinical trials driven by experimental evidence and by exploring new anti-cancer therapeutics. Moreover, he encouraged the physicians running the clinical trials to collaborate with research scientists to elucidate the individual patient’s response to therapy.

Over the past 30 years, José was involved in the development of at least twelve FDA-approved anti-cancer agents. He published around 500 peered review publications and was in a member of Editorial advisory boards of top journals such as Journal of Clinical Oncology and Cancer Cell. He co-founded and led as Editor in Chief Cancer Discovery, now a reference source for the oncology community. José received many awards and served as the president of ESMO and the AACR.

As a mentee, I sincerely appreciate the time and effort José invested in me and in all his mentees, and I cherish his sincere willingness to help in developing the careers of many translational researchers and oncologists. I will always remember his generosity, remarkable sense of humor, and genuine passion for science.

It is with tears and deep sorrow that we extend our deepest condolences to José’s family and to all those who were close to him.

Rest in peace my dear friend and mentor.

*Moshe Elkabets Ph.D.*

Dr. Baselga thank you very much for inspiring future generations with your visionary leadership in translational and medical oncology.

*Giovanni Blandino MD.*
